# Time perception changes in stroke patients: A systematic literature review

**DOI:** 10.3389/fneur.2022.938367

**Published:** 2022-07-19

**Authors:** Pedro Coelho, Joana Amado Rodrigues, Pedro Nascimento Alves, Ana Catarina Fonseca

**Affiliations:** ^1^Serviço de Neurologia, Departamento de Neurociências e Saúde Mental, Hospital de Santa Maria, Centro Hospitalar Universitário Lisboa Norte, Lisboa, Portugal; ^2^Centro de Estudos Egas Moniz, Faculdade de Medicina, Universidade de Lisboa, Lisboa, Portugal; ^3^Clínica Universitária de Neurologia, Faculdade de Medicina, Universidade de Lisboa, Lisboa, Portugal; ^4^Laboratório de Estudos de Linguagem, Centro de Estudos Egas Moniz, Faculdade de Medicina, Universidade de Lisboa, Lisboa, Portugal; ^5^Instituto de Medicina Molecular João Lobo Antunes, Faculdade de Medicina, Universidade de Lisboa, Lisboa, Portugal

**Keywords:** time perception, chronotaraxis, stroke, scalar expectancy theory, striatal beat frequency, behavioral neuroscience

## Abstract

**Introduction:**

Time perception comprises the subjective experience of passing of time and of the duration of an event. Although already described in some neurological and psychiatric conditions, there is a paucity of details regarding this neurocognitive change in stroke patients. We aimed to describe time perception dysfunction in stroke patient.

**Methods:**

We performed a systematic review of the literature in Pubmed, PsycInfo and EMBASE including manuscripts from their inception until December 2020. We collected data regarding the type of time perception that was detected, type of stroke, most common location of lesions, evaluation tests that were used and time of evaluation after stroke onset.

**Results:**

A total of 27 manuscripts were selected, concerning a total of 418 patients (*n* = 253 male; 60.5%). Most manuscripts (*n* = 21) evaluated patients with ischaemic lesions (*n* = 407; 97.4%). The majority referred to evaluations between 2 months and seven years after stroke. Underestimation in temporal evaluation in sub- and supra-second was the most common dysfunction (*n* = 165; 41.7%). Overestimation of time (*n* = 116; 27.8%) and impaired time interval comparison (*n* = 88; 22.2%) were also found. Most patients had right hemisphere lesions (*n* = 219 patients; 52.4%). Common reported lesion locations included the thalamus, insula, basal ganglia, dorsolateral prefrontal cortex, parietal cortex including supramarginal, angular gyrus and right inferior parietal cortex and cerebellum.

**Conclusion:**

There are multiple stroke locations associated with time perception dysfunction, which highlights the complex system involved in time perception. There is still scarce knowledge about specific time perception deficits after stroke. Most studies rely in psychometric analysis without clear clinical and functional translation, namely regarding impact on daily activities.

## Introduction

Time perception comprises the subjective experience of the passing of time and the duration of an event (1). This cognitive ability is crucial for our life, including our interaction with others and the external world, and results from a whole integration of environmental changes and cognitive processes (1, 2).

Diverse brain structures have been associated with time perception such as the dorsolateral and inferior prefrontal cortex, supplementary motor area, anterior cingulate gyrus, inferior parietal cortex, basal ganglia, cerebellum and hippocampus (1, 3). These structures are involved in diverse types of time perception. Different ranges of time intervals (milliseconds or seconds-minutes) rely on different mechanisms. T sub-second intervals are coordinated by automatic mechanisms integrated in sensory and motor action plans (4, 5) and supra-second intervals require greater cognitive control, depending on attentional and working-memory processes (4–6). Moreover, the contribution between these interconnected structures is flexible and depends not only on the duration of the time interval to be assessed by the brain but also on the type of cognitive task and the stimulus modality used for marking time (4).

Time perception can also be divided in tasks that require explicit or implicit timing (7, 8). The former occurs when an explicit estimation of time interval duration or active time pattern recognition is perceived by sensory modalities or through a motor response (e.g., learning a rhythm and being able to replicate it afterwards); the latter occurs when a regular temporal pattern is also perceived by sensory stimuli or motor responses but is then used for a non-temporal goal (e.g., understanding if it is safe to cross the street while a car is approaching us) (7, 8).

Different theories and models have tried to explain how these diverse brain regions and networks develop a unified time perception mechanism (9, 10). These models are generally classified as dedicated and intrinsic time perception models (6). Dedicated models believe that centralized brain circuits are responsible for time perception across different modalities, tasks, and scales of time (8). Scalar Expectancy Theory and Striatal Beat-Frequency are paradigmatic examples of dedicated models (9, 11).

Intrinsic models like state-dependent networks (12), propose that time perception is an intrinsic quality of most neural circuits and timing emerges from *online* changes in the dynamics of neurons and neural circuits (8).

Other researchers suggests that time perception mechanisms are derived from a hybrid model with a partially distributed timing mechanism, which includes a core network - the cortico-thalamic-basal ganglia (CTBG) circuit - and areas that are selectively engaged by different behavioral contexts come into play in different contexts (10).

Time perception is also influenced by patients' age and mood (13–15). Several studies reported reduced timing abilities in older adults (14) and age-related changes in time perception can be a potential cognitive marker for neurodegenerative diseases, although there is still conflicting evidence regardingthis subject (14, 16, 17). Depressed and suicidal patients also present changes in time perception, namely the experience of time dilation or slower time flow (15). The effect of patients' literacy in time perception is less clear (13, 16).

Time perception deficits have already been characterized in various neurological and psychiatric conditions including Parkinson's disease, schizophrenia, depression, autism and attention deficit and hyperactivity disorder (1, 7). However, there is limited information about disturbed perception of time after stroke and of how this may impact patients' functional independence.

The aim of this systematic literature review was to characterize time perception dysfunction in stroke patients, namely the type of time perception dysfunction associated with stroke lesions, its anatomical locations, and the impact of this disability on patients' outcome.

## Methods

For the purpose of this systematic literature review, we followed the Meta-Analysis of Observational Studies in Epidemiology (MOOSE) (18) and the Preferred Reporting Items for Systematic Reviews and Meta-Analyses (PRISMA) guidelines (19).

### Information sources and search strategy

A systematic literature review was performed by accessing the following databases: Pubmed, EMBASE and PsycInfo. We included articles from the databases inception until December 2020. Keywords used were time perception ([MeSH Terms] AND stroke [MeSH Terms]) and “(temporal processing [MeSH Terms] AND stroke [MeSH Terms]).” The reference lists of potential eligible studies/selected studies reference lists were crosschecked for additional studies.

### Eligibility criteria

We considered published observational studies reporting time perception dysfunction in stroke patients. Any observational study design was accepted, including case reports independently of the number of patients included. Articles in English, French, Spanish, Italian and Portuguese were accepted.

### Study selection

The title and abstract of potential eligible studies were screened by three authors (PC, JR, and AF). Selection of the included papers was performed independently by the three authors; disagreements were solved by consensus. Full texts of potentially eligible studies were obtained. Duplicated studies were removed.

### Data collection process and data items

For the selected studies we extracted the following data: demographic data (number of patients, gender, age), stroke type (ischaemic or haemorrhagic), stroke topography, time between stroke and evaluation, brain image modality used for lesion evaluation, protocol used for time perception evaluation, results from time perception evaluation, neuropsychological evaluation protocol, neuropsychological evaluation results, neurological deficits associated with stroke lesion, follow-up evaluation. Data were extracted from reports to predesigned tables, independently by two authors.

### Time perception evaluation protocol

The different time perception evaluation protocols applied in the studies were classified in subtypes according to Grondin (2010) (20). This classification divides general paradigms protocols in prospective timing (participants know in advance that they will perform a task in which time duration will be asked after the task) and retrospective timing (participants receive no prior warning). In retrospective timing paradigms, time can be evaluated with verbal estimation and interval reproduction protocols. In prospective timing paradigms, time can be evaluated with verbal estimation, interval reproduction, interval production and interval comparison.

Time perception evaluation was defined as acute when patients were evaluated within 1 month after stroke.

### Quality of selected studies

The selected studies were submitted to quality evaluation following recommended guidelines (Newcastle Ottawa Quality Assessment Scale for case control studies and Murad MH et al. guidelines for case series and case reports) and were independently analyzed by three authors (PC, JR, and AF). Disagreements were solved by consensus. The quality evaluation is available in the Supplementary Material.

### Data presentation and statistical analysis

For each study we displayed the various variables collected with absolute numbers and corresponding percentages. A descriptive statistical analysis of the data collected was performed with collected data.

## Results

**Figure 1:**
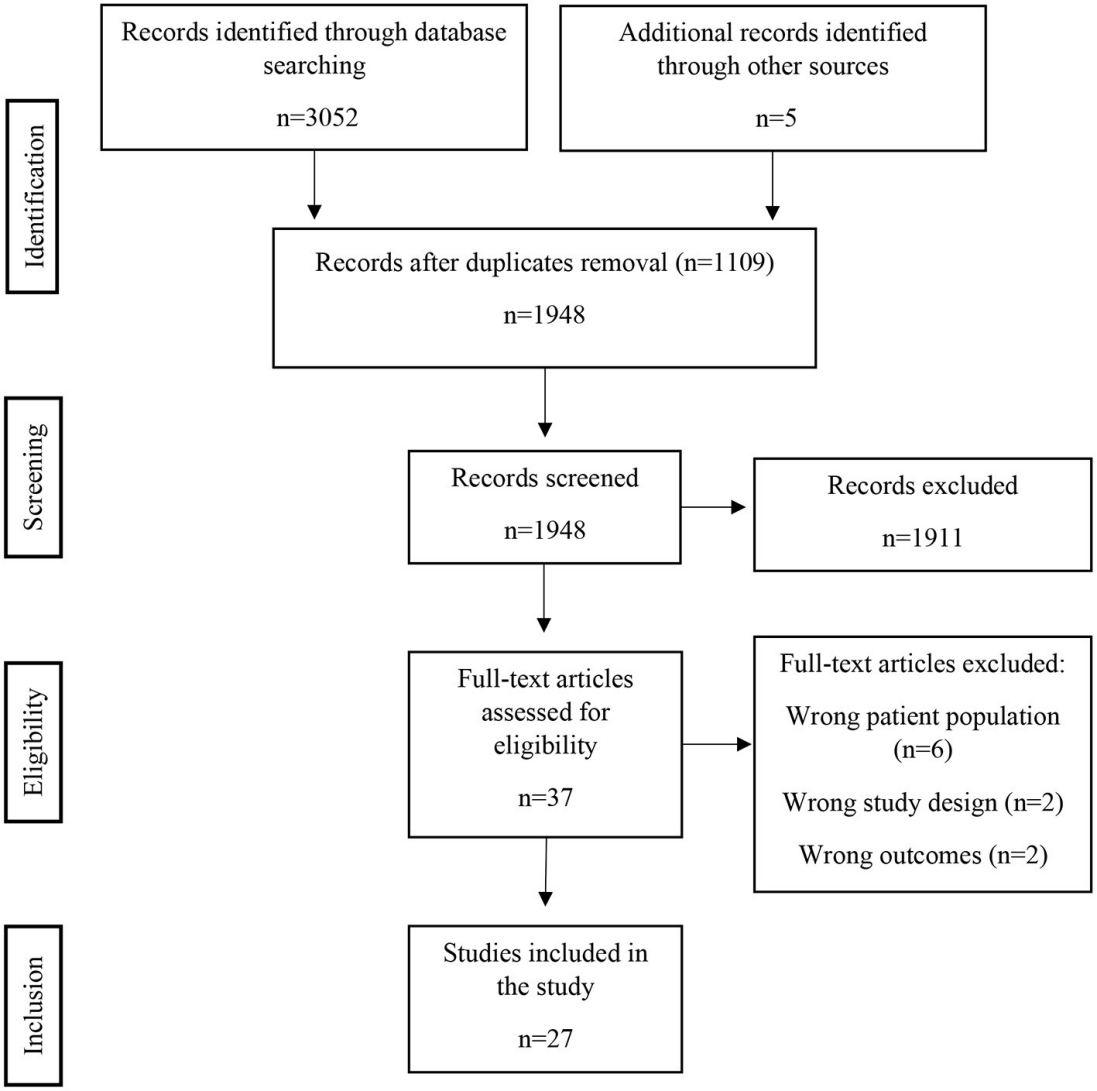
PRSIMA 2020 flow diagram. There were 1,911 records excluded after screening, mostly because the subject addressed was not time perception (e.g., time in context of neuropsychological experiments not directly related to time perception, neurophysiology and time response of neurons, etc.). The full-text articles were excluded due to wrong patient population (patients with neurological diseases other than stroke – e.g., brain tumor), wrong study design (mainly focusing in other cognitive dysfunctions besides time perception or where time perception is due to another primarily cognitive dysfunction) or wrong outcomes (differences in motor tasks that occur due to time perception dysfunction but that do not translate into a specific time perception deficit).

A total of 3,057 articles were found (PRISMA flowchart in Figure 1); after removal of duplicates, a total of 1,943 articles were initially considered for title and abstract screening. Thirty-seven articles were selected for full-text screening. Finally, 27 studies met the final inclusion criteria and were included, concerning a total of 418 patients (Table 1). Among these studies, there were 24 case-control studies, 2 case reports and 1 case series.

**Table 1:** Characterization of demographic and clinical information.

**Number of patients**	**418**
**Age, mean ±standard deviation**	59.25 ± 11.87
**Age range, min-max**	35-88
**Male, *n* (%)**	253 (60.5%)
**Stroke type (%)**	
Ischaemic	407 (97.4%)
Haemorrhagic	11 (2.6%)
**Brain image modality**	
CT, *n* (%)	85 (21.5%)
MRI, *n* (%)	188 (45%)
Not specified (CT or MRI), *n* (%)	145 (34.7%)
**Stroke laterality**	
Right hemisphere, *n* (%)	219 (52.4%)
Left hemisphere, *n* (%)	199 (47.6%)

Most patients were male (*n* = 253; 60.5%) and patients' mean age was 59.13 ± 11.82, ranging between 35 and 88 years. There were no data regarding the patients' number of years of formal education. Most manuscripts (*n* = 21) evaluated patients with ischaemic lesions; the remaining 6 studies had ischaemic and haemorrhagic strokes, with haemorrhagic stroke comprising only 11 patients. Five manuscripts reported results from acute evaluations (*n* = 93; 22.2%), while the majority (*n* = 309; 73.9%) referred to evaluations between 1 month and 7 years after stroke onset. Only three studies were prospective, with follow-up time of 3 and 6 months.

Brain MRI was the most reported brain image modality for lesion evaluation. Right hemisphere lesions were more frequent (*n* = 219 patients; 52.4%); only 5 patients had bilateral lesions. Twenty-two studies focused on supratentorial lesions, 3 had infratentorial lesions and 2 had both supra and infratentorial lesions.

The location of lesions reported included the thalamus, insula, basal ganglia, dorsolateral prefrontal cortex, parietal cortex including supramarginal, angular gyrus and right inferior parietal cortex and cerebellum (data concerning more specific detail regarding lesion location is shown in Table 2 and in Supplementary Table 1).

**Table 2:** Characterization of stroke location.

**Supratentorial lesions**, *n* (%)	
Cortical and subcortical lesions involving thalamus and basal ganglia, *n* (%)	293 (70.1%)
Cortical and subcortical lesions excluding thalamus and basal ganglia, *n* (%)	41 (9.8%)
Thalamic lesions, *n* (%)	8 (1.9%)
Basal ganglia lesions, *n* (%)	1 (0.2%)
Insular lesions, *n* (%)	21 (5%)
**Infratentorial lesions**, *n* (%)	
Cerebellar lesions, *n* (%)	54 (12.9%)

**Figure 2:**
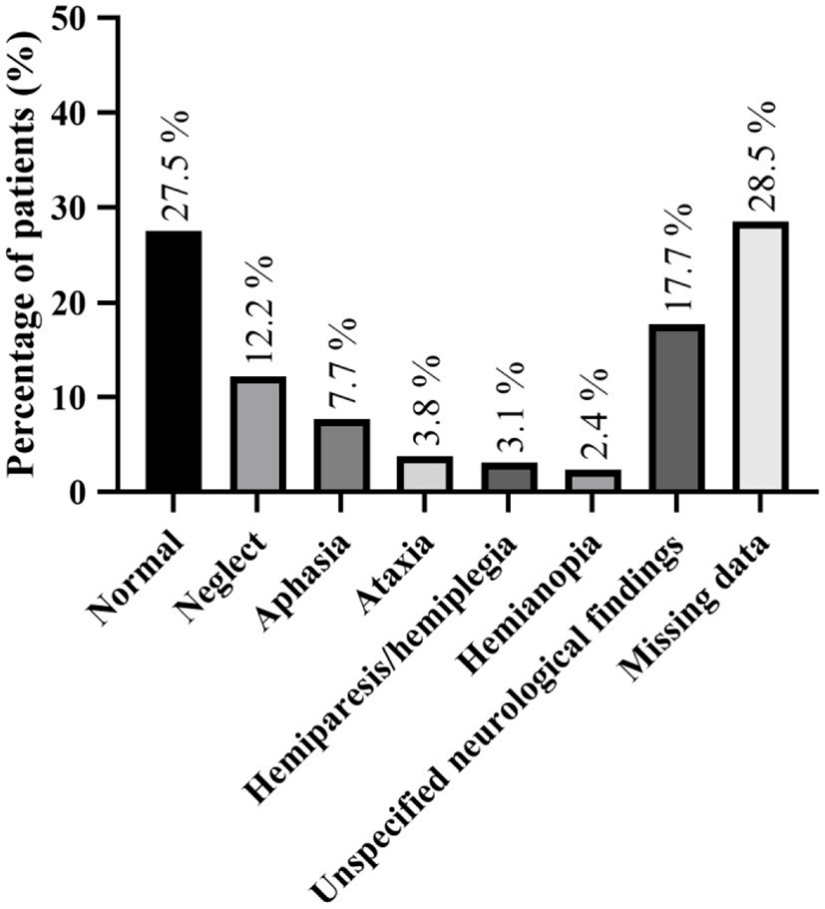
Detailed neurological examination findings; more than one neurological defect could be found in some patient.

Neurological examination details were present in 19 studies (depicted in Figure 2 and in Supplementary Table 1). The most common reported concomitant neurological deficits were neglect (*n* = 51, 12.2%) and aphasia (*n* = 32, 7.7%). In 115 patients (27.5%), the neurological examination was described as normal. However, a significant number of studies did not provide any details about neurological examination (*n* = 119; 28.5%).

**Figure 3:**
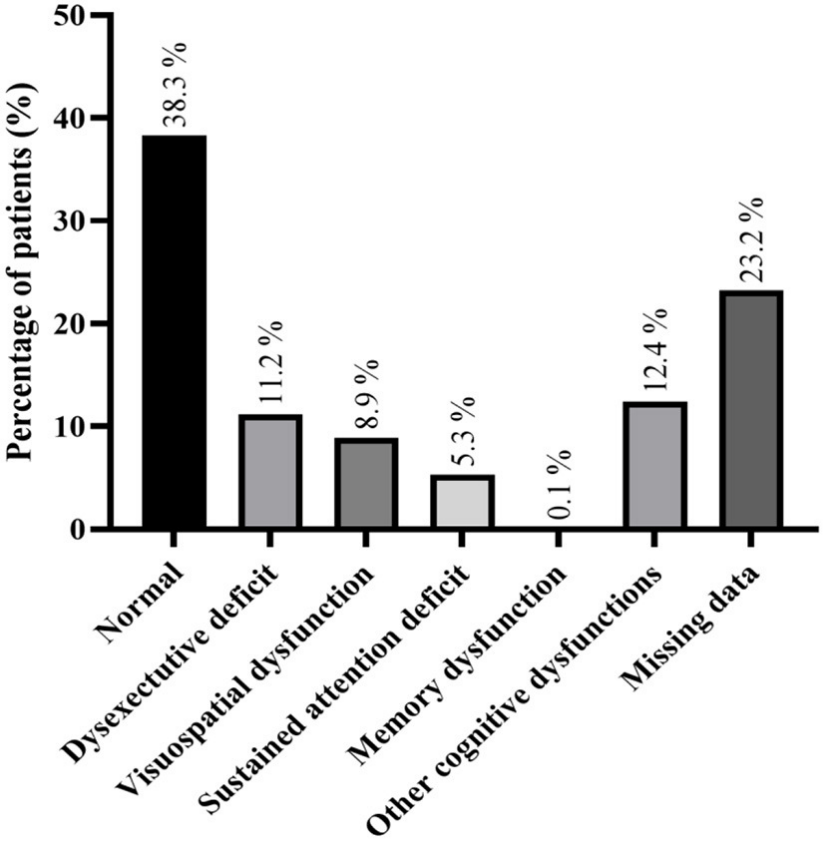
Detailed neuropsychological examination; more than one neuropsychological dysfunction could be found in some patient.

Information about neuropsychological evaluation was present in 22 studies and 168 (40.2%) patients had a normal neuropsychological evaluation (further details in Figure 3 and Supplementary Table 1). The most common neuropsychological defect was dysexecutive deficits, occurring in 47 patients (11.2%).

Most studies (*n* = 17) applied temporal evaluation protocols referring to prospective timing in sub- and supra-second task (2 seconds until 90 seconds), including visual and auditory stimuli time interval comparison, visual and auditory stimuli time estimation and time reproduction. Retrospective timing protocols involved mostly time duration estimation.

**Figure 4:**
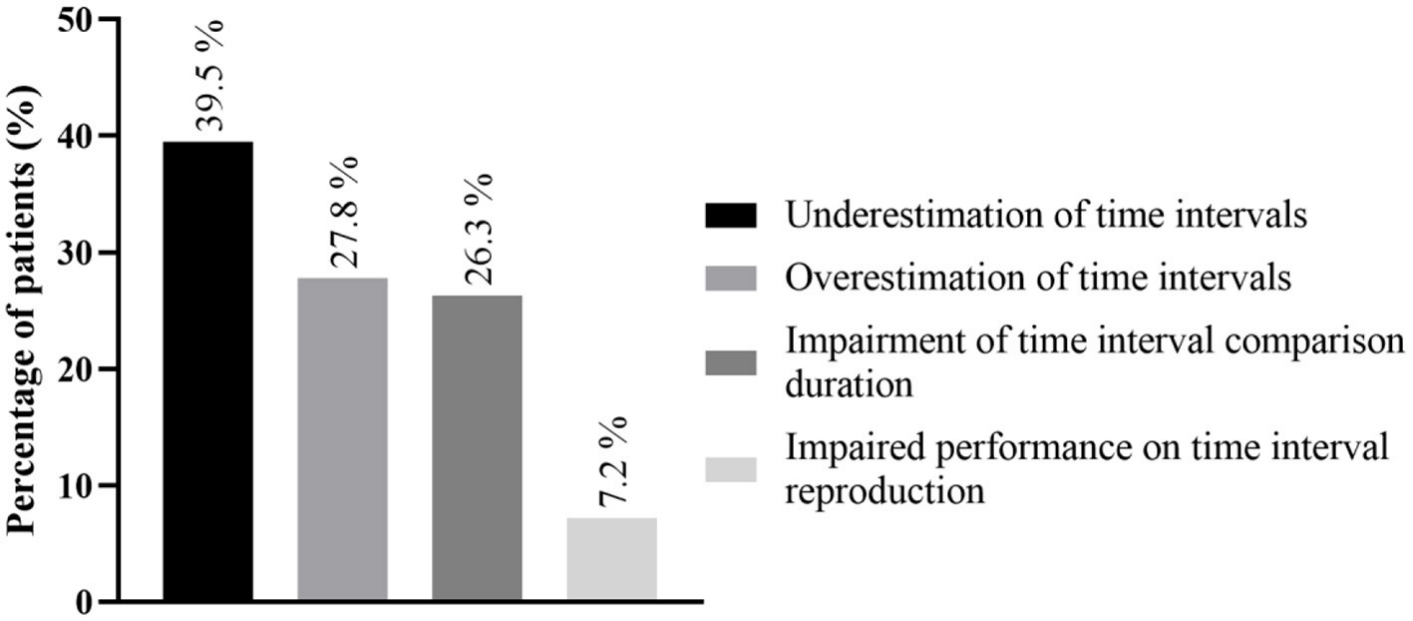
Main time perception dysfunctions; detailed information regarding all time perception deficits is found in Supplementary Table 2. More than one time perception defect could be found in the same patient.

The most common dysfunction of time perception that was found was underestimation in the temporal evaluation of sub- and supra-second intervals, reported in 12 studies concerning 165 patients (39.5%). The other time perception dysfunctions found are depicted in Figure 4, Table 3 and Supplementary Table 2.

**Table 3:** Lesion topography and respective associated time perception deficits.

**Lesion location**	**Time perception deficit associated**
Cerebellar hemisphere	Underestimation, overproduction of time intervals between 2–12 sec; worse in left cerebellar hemisphere lesions (21) Impaired interval reproduction in the seconds range (22, 23) Impairment in perceptual timing (24, 25)
Right supramarginal gyrus (including involvement of right basal ganglia, insula and superior temporal gyrus)	Underestimation of time duration for intervals of 15–60s (worse on neglect patients) (26)
Prefrontal cortex	Increased difference thresholds in time perception tasks (24, 25); worse performance with dual tasking (divided attention) (25) Underestimation of time interval comparison (24) Underestimation of time duration for long time durations (>90 s) (27) Impairment in time interval comparison (right prefrontal cortex) (28)
Right temporal-occipital region (including thalamus and posterior arm of internal capsule)	Overestimation of time duration of stimuli on left hemifield (29)
Diffuse right hemispheric lesion	Slower timing responses in temporal order judgment (neglect patients) (30) Impairment in clock-time estimation and interview duration estimation (31) Overestimation of time intervals (32–35) Underproduction of time intervals (32) Undereproduction of time intervals (33) Underestimation of duration of time intervals (36, 37) Impairment in time interval comparison (28, 34, 35)
Diffuse left hemispheric lesion	Overestimation, underproduction of time intervals (32) Overestimation of time intervals (33) Underestimation of time intervals (33, 37)
Thalamus	Temporal disorientation, impaired estimation of day time and impaired estimation of the examination duration (38–40) Impairment of time interval reproduction (39, 41) Impaired estimation of time interval duration (40, 41)
Basal ganglia	Overestimation in time interval comparison in 300 ms range (42) Impairment on time reproduction in 50 ms range (42)
Right insula	Underestimation of time duration for short time intervals (43) Impairment in perceptual timing (43)
Parietal lobe (left medial, lateral and superior parietal lobe, intraparietal sulcus with extension to temporal region)	Underestimation of time intervals with task-irrelevant numerical stimuli; near chance performance of interval comparison of visual stimuli with numeral stimuli (36)
Right temporoparietal junction	Underestimated leftward motion duration in comparison with rightward motion duration when patients had impaired spatial orientation (44)
Right parietal lobe (including fronto-parietal subcortical white matter)	Underestimation of time duration on different protocols in 15–60 s interval (45)
Right inferior parietal cortex (including rolandic operculum and posterior middle temporal gyrus)	Impairment in prospective timing (46)
Right superior temporal gyrus, white matter posterior to the insula and posterior insula	Impairment in retrospective timing (46)
Posterior supramarginal white matter	Impaired time estimation (33)
Frontal lobe	Impairment in perceptual timing (47)
Posterior parietal lobe	Impairment in perceptual timing (47)
Right precentral, middle frontal and inferior frontal gyrus	Impairment in perceptual timing under and above 1 second (47)
Left basal ganglia, superior and middle temporal lobe and hippocampus	Impairment in perceptual timing under 1 second (47)

*Times deficits regarding production relate to the ability of patients being able to produce a time interval without any kind of referential or previous cue, only using an internal representation of time; time deficits regarding reproduction relate to the ability of patients being able to reproduce faithfully a previously shown/heard time interval. Impairments in perceptual timing concern abnormal capability in time accuracy in different modalities, when compared to controls (e.g., more prone to under and overestimation or under and overproduction than healthy controls)*.

No study evaluated the impact of time perception dysfunction on patients' functionality, namely its impact on daily day activities.

## Discussion

This study represents the first systematic review of time perception deficits in stroke patients, to our knowledge. Time perception deficits have already been characterized in different psychiatric and neurological conditions, but not in stroke, which is one of the most common neurological diseases (48).

Overall, we found a low number of articles reporting time perception deficits in stroke patients. Time perception deficits in stroke are probably underestimated and most of the times not diagnosed or reported since these deficits are not methodically evaluated on routine clinical practice.

We found time perception deficits to be slightly more common in strokes affecting the right hemisphere (219 vs. 199 in the left hemisphere). Numerous locations were associated with time perception deficits, namely the thalamus (38–41), insula (43), basal ganglia (42), cerebellum (21–25) and cortical structures (dorsolateral prefrontal cortex (24, 25, 27, 28, 32, 36, 44, 47), parietal cortex (32, 36, 44–47), including supramarginal (26, 28, 33) and angular gyri (28), temporal cortex (26, 32, 36, 46, 47). These locations correspond to those commonly reported in time perception studies with functional brain MRI in healthy participants (5, 49). Also, the lesions' location that we found were consistent with previews reviews in time perception deficits (4, 7, 50, 51).

The most common defect found in our work was underestimation in temporal evaluation, which implies a subjective acceleration of the sense of the passage of time, involving 165 patients (41.7%). Other common defects were overestimation of time (*n* = 116, 27.8%) and impaired time interval comparison (*n* = 88, 22.2%).

The great diversity in terms of locations involved and the overlapping time perception deficits across these different locations demonstrate the complexity of time perception mechanisms and how, more than a lesional approach, time perception is the end result of diverse systems finely arranged.

Cortical and subcortical structures are implied in both sub-second and supra-second tasks, although subcortical areas are predominantly involved in sub-second intervals (more dependent on sensory and automatic processes) and supra-second intervals rely more on activation of cortical areas (4, 52).

Between the multiple cortical areas related to time perception, including supplemental motor area, inferior parietal cortex and superior temporal cortex, predominantly in the right hemisphere (5, 51), the dorsolateral prefrontal (DLPFC) is considered the region most involved in time perception (1), particularly the right prefrontal cortex (3). One of the most important functions of DLPFC is its role in sustained attention to the time interval or working memory components, acting as an “accumulator”, storing information about a passing time interval and making it the working memory component of a hypothesized internal clock (3). The right inferior parietal cortex also has a role in these attentional processes as part of a right prefrontal inferior parietal network that is engaged in duration estimation, particularly for longer durations (51). The left parietal and premotor cortex have been implied in temporal predictability (7). Furthermore, the parietal cortex seems crucial when time information has to be processed together with spatial information, for both sub- and supra-second time intervals (4). The right temporal lobe is responsible for retrieval and comparison of temporal duration representations (51).

The supplemental motor area (SMA) is another core brain region involved in timing, sharing a central and ubiquitous role together with basal ganglia in both motor and perceptual timing (7). SMA also functions as an online timing (or accumulation) of a stimulus duration that is currently unfolding in time (7). These cortical structures are also connected to the thalami and striatal structures in a thalamo-cortical-striatal network that is involved and activated in temporal tasks (9).

The impact of stroke lesions in the thalamus support the role that this structure has on time perception mechanisms by being a part of the circuitry involving the cerebellum, basal ganglia and cerebral cortex (10), namely the mediodorsal and anteromedial nuclei, that constitute the “limbic thalamic nuclei” (53). These structures are probably essential for memory formation that translates into the notion of time intervals (41). Previous studies suggest that these nuclei have important projections, namely dorsolateral prefrontal cortex and receive basal ganglia projections (54). Moreover, lesions in the mediodorsal nuclei are associated with a decrease in cerebral blood flow in cortical areas, most notably the dorsolateral prefrontal cortex (39, 54), an essential region in time perception circuits.

The insula is also involved in the time perception circuit (43, 55). Both the anterior portion and posterior part of the insula have been implicated in time perception tasks, with the posterior part being more involved in the encoding phase of time perception tasks and the anterior part more involved in the reproduction phase where an explicit judgment of duration is made (49, 56). It is believed that insula has an important role for self-notion of time, by integrating received signals in dorsal posterior insula that are processed in a posterior-to-anterior progression and cumulate in the anterior insula, creating the experience of duration.

The cerebellum has been widely implicated in time perception and reproduction, especially in sub-second intervals and in motor timing tasks (4, 7, 21). It has been proposed that timing in cerebellum relies on the spatiotemporal dynamics of the granule cell population, dependent of the influence of cerebellar input and the internal state of the cerebellar network (57). This changing network of cells inherently encodes time, which then is interpreted by the Purkinje cells (57).

There seems to be a functional specialization for timing in discrete zones of the cerebellum, with studies showing that the more superior (23) and lateral parts (22) are being involved in time perception. Moreover, a number of functional imaging studies in time perception tasks have reported exclusive activation of the left cerebellar hemisphere (21). Lateral cerebellum contains dentate nuclei, which project to the premotor, dorsolateral prefrontal (DLPFC) and parietal cortices (23), regions that are also involved in timing mechanisms. The disruption of the cerebellar-DLPFC network could also explain the impact of cerebellar lesions in the long-term time perception dysfunction. The cerebellum is also involved in temporal prediction by being part of left premotor-cerebellar-parietal circuits capable of prediction, as well as working memory tasks, essential for supra-second intervals (58).

Basal ganglia are fundamental structures concerning time perception in the range of milliseconds to several seconds (3, 59). One of the reasons for this key role in timing could be explained by the striatal beat frequency theory, a neurobiological time perception model that postulates that representation of time is influenced by the striatum's ability to detect similar patterns of cortical and thalamic oscillations, and then synchronize neural firing in response to different requirements of time perception (3, 9). Distortions in time perception with dopamine agonists and antagonists confirm the central role of basal ganglia in timing (1, 3, 59, 60).

The sample of patients that was evaluated was heterogeneous in terms of age range, neurological examination findings and neuropsychological evaluation. A large variation in patients' age could limit some of the findings in this study, since older people can have impaired time perception, with a subjective slowing of their internal clock (13, 17). Nevertheless, most studies relied on a case-control study design, which hampers some of the possible limitations of wide age variation in the studies.

Clear information regarding the neurological examination and neuropsychological evaluation were missing, which is another limitation that could influence the results. Neurological deficits and neurodegenerative diseases contribute to time perception dysfunctions (17).

Neglect was the most common neuropsychological deficit accompanying time perception deficits. The fact that the right parietal cortex is both involved in time perception and spatial and attentional networks could explain this finding. Although more studies are necessary regarding the interplay between neglect and time perception deficits, there seems to be some evidence that deficits of timing behavior are likely to be more severe in patients with right hemisphere damage and most severe in patients with neglect following right parietal damage (45).

Several time perception protocols were used through the different studies analyzed, which limits comparisons between different studies and broader conclusions. Most of the protocols relied on complex paradigms of time perception evaluation, which also highlights how difficult it is to evaluate time perception in a systematic manner. These protocols are laborious and time consuming, requiring very precise tools not readily available and gathering results not always easy to understand. The characteristics associated to these testing modalities hamper the possibility of widespread and systematic evaluation for most patients. Furthermore, the nature of psychometric testing does not depict the patients' complaints or the daily impact of the time perception deficits, which restricts the conclusions that these time perception deficits have on patients' life and how to further develop rehabilitation specifically tailored to address the problems that arouse as consequence of time perception dysfunction. This issue has already been raised in previous studies (14). In this regard, the QUEST-R questionnaire presented in Trojano et al. (46) study is a tool that permits the evaluation of patients in a simple and methodical way and allows the f translation of results into a more practical context.

Besides the points discussed above, other major limitation in this work is the low number of articles found in our systematic review. Most studies were retrospective, which also limits follow-up information about the maintenance of these time perception deficits and could be a possible source of bias.

Our findings give some information regarding the most common time perception deficits found in stroke patients. This information will hopefully encourage a more frequent systematic evaluation of time perception deficits in stroke patients, namely in the acute phase. The impact that these changes can have in the functional independence and rehabilitation program of patients is still unknown. Future prospective studies are needed. An increased awareness for this specific type of deficits will help to develop new standardized beside protocols.

## Conclusion

We found a low number of articles reporting time perceptions changes in stroke which may be due to underreporting or underdiagnosis. The most common time perception deficit was underestimation of time, followed by overestimation of time.

A methodical search of this dysfunction in stroke patients and development of standardized bedside protocols is needed for further developments in this area of research.

## Data availability statement

The original contributions presented in the study are included in the article/supplementary material, further inquiries can be directed to the corresponding author/s.

## Author contributions

Material preparation, data collection, and analysis were performed by PC, JR, and AF. The first draft of the manuscript was written by PC. All authors commented on previous versions of the manuscript, contributed to the study conception and design, read, and approved the final manuscript.

## Conflict of interest

The authors declare that the research was conducted in the absence of any commercial or financial relationships that could be construed as a potential conflict of interest.

## Publisher's note

All claims expressed in this article are solely those of the authors and do not necessarily represent those of their affiliated organizations, or those of the publisher, the editors and the reviewers. Any product that may be evaluated in this article, or claim that may be made by its manufacturer, is not guaranteed or endorsed by the publisher.
