# Supplementary material for: Time perception changes in stroke patients: A systematic literature review
Source: Front Neurol. 2022 Jul 19;13:938367. doi: 10.3389/fneur.2022.938367 (PMC9343772; doi:10.3389/fneur.2022.938367)
Supplement: Supplementary file 1 [file Data_Sheet_1.docx]

**Appendix**

**Appendix 1: Quality evaluation according to Newcastle-Ottawa Scale (NOS) for assessing the quality of nonrandomised studies in meta-analyses**

| **Article** | Gooch et al.^16^ | Harrington et al.^17^ | Mangels et al. ^19^ | Casini et al. ^44^ | Basso et al. ^21^ | Bonato et al.^22^ | Morin et al.^23^ | Kumral et al. ^24^ | Coslett et al. ^25^ |
| --- | --- | --- | --- | --- | --- | --- | --- | --- | --- |
| **Selection** | | | | | | | | | |
| 1. Is the case selection adequate? | a) | a) | a) | a) | a) | a) | a) | a) | a) |
| 1. Repressentiveness of the cases | b) | b) | b) | b) | b) | a) | a) | a) | b) |
| 1. Selection of Controls | a) | a) | a) | a) | a) | a) | a) | c) | c) |
| 1. Definition of Controls | a) | a) | a) | a) | a) | a) | a) | a) | a) |
| **Comparability** |  |  |  |  |  |  |  |  |  |
| 1. Comparability of cases and controls on the basis of the design or analysis | a) | a) | a) | a) | a) | a) | a) | 0 | a) |
| **Exposure** |  |  |  |  |  |  |  |  |  |
| 1. Ascertainment of exposure | c) | c) | c) | c) | c) | c) | c) | c) | c) |
| 1. Same method of ascertainment for cases and controls | a) | a) | a) | a) | a) | a) | a) | a) | a) |
| 1. Non-Response rate | a) | a) | a) | a) | a) | a) | a) | a) | a) |

Table 3 - Quality evaluation according to Newcastle-Ottawa Scale (NOS) for assessing the quality of nonrandomised studies in meta-analyses. Based on Wells GA, Shea B, O'Connell D, Peterson J, Welch V, Losos M, et al. The Newcastle-Ottawa Scale (NOS) for assessing the quality of nonrandomised studies in meta-analyses. The Ottawa Hospital Research Institute; 2014. http://www.ohri.ca/programs/clinical_epidemiology/oxford.asp

| **Article** | Coslett et al.^26^ | Cappelletti et al.^27^ | Kaski et al.^28^ | Harrington et al.^29^ | Mella et al.^30^ | Rubia et al.^31^ | Trojano et al.^32^ | Gooch et al.^33^ | Merrifield et al.^34^ |
| --- | --- | --- | --- | --- | --- | --- | --- | --- | --- |
| **Selection** | | | | | | | | | |
| 1. Is the case selection adequate? | a) | a) | a) | a) | a) | c) | a) | a) | a) |
| 1. Repressentiveness of the cases | b) | a) | b) | b) | a) | b) | a) | a) | b) |
| 1. Selection of Controls | c) | c) | c) | c) | c) | c) | b) | c) | a) |
| 1. Definition of Controls | a) | a) | a) | b) | a) | a) | a) | a) | a) |
| **Comparability** |  |  |  |  |  |  |  |  |  |
| 1. Comparability of cases and controls on the basis of the design or analysis | 0 | a) | 0 | a) | 0 | 0 | a) | a) | 0 |
| **Exposure** |  |  |  |  |  |  |  |  |  |
| 1. Ascertainment of exposure | c) | c) | c) | c) | c) | c) | c) | c) | c) |
| 1. Same method of ascertainment for cases and controls | a) | a) | a) | a) | a) | a) | a) | a) | a) |
| 1. Non-Response rate | a) | a) | a) | a) | a) | a) | a) | a) | a) |

Table 3 – (continued)

| **Article** | Low et al.^35^ | Mole et al.^36^ | Danckert et al.^37^ | Koch et al.^38^ | Calabria et al.^41^ | Montalembert et al.^42^ |
| --- | --- | --- | --- | --- | --- | --- |
| 1. Is the case selection adequate? | a) | a) | a) | a) | a) | a) |
| 1. Repressentiveness of the cases | a) | b) | b) | b) | a) | a) |
| 1. Selection of Controls | a) | c) | c) | c) | c) | c) |
| 1. Definition of Controls | a) | a) | a) | a) | a) | a) |
| **Comparability** |  |  |  |  |  |  |
| 1. Comparability of cases and controls on the basis of the design or analysis | a) | 0 | 0 | 0 | b) | b) |
| **Exposure** |  |  |  |  |  |  |
| 1. Ascertainment of exposure | c) | c) | c) | c) | c) | c) |
| 1. Same method of ascertainment for cases and controls | a) | a) | a) | a) | a) | a) |
| 1. Non-Response rate | a) | a) | a) | a) | a) | a) |

Table 3 – (continued)

**Appendix 2: Quality evaluation according to Murad et al. for assessing the quality of case reports**

| **Article** | Malapani et al. ^43^ | Hayashi et al.^39^ | Lee et al.^40^ |
| --- | --- | --- | --- |
| 1. Does the patient(s) represent(s) the whole experience of the investigator (centre) or is the selection method unclear to the extent that other patients with similar presentation may not have been reported? | No | No | No |
| 1. Was the exposure adequately ascertained? | Yes | Yes | Yes |
| 1. Was the outcome adequately ascertained? | Yes | Yes | Yes |
| 1. Were other alternative causes that may explain the observation ruled out? | Yes | Yes | Yes |
| 1. Was there a challenge/rechallenge phenomenon? | NA | NA | NA |
| 1. Was there a dose–response effect? | NA | NA | NA |
| 1. Was follow-up long enough for outcomes to occur? | NA | NA | NA |
| 1. Is the case(s) described with sufficient details to allow other investigators to replicate the research or to allow practitioners make inferences related to their own practice? | Yes | Yes | No |

Table 4 - Quality evaluation of case series and case reports ; NA – not applied. Based on Murad MH, Sultan S, Haffar S, Bazerbachi F. Methodological quality and synthesis of case series and case reports. BMJ Evidence-Based Medicine 2018; 23:60-63. http://dx.doi.org/10.1136/bmjebm-2017-110853.
