# Supplementary material for: Time perception changes in stroke patients: A systematic literature review
Source: Front Neurol. 2022 Jul 19;13:938367. doi: 10.3389/fneur.2022.938367 (PMC9343772; doi:10.3389/fneur.2022.938367)
Supplement: Supplementary file 2 [file Table_1.docx]

**Supplemental Table 1** - Studies included in systematic review. LH -left hemisphere, RH – right hemisphere; BADS - Behavioural Assessment of the Dysexecutive Syndrome, D-KERS - Delis–Kaplan Executive Function System, FAB – Frontal Assessment Battery, MMSE – Mini Mental Status Exam, MoCA – Montreal Cognitive Assessment, NART-IQ – National Adult Reading Test, RBMT- III - Rivermead Behavioural Memory Test, RMT – Recognition Memory Test RPM – Raven Progressive Matrices, RWL - Rey Word List, TMT – Trail Making Test, ToL – Tower of London, WAIS-IV - Wechsler Adult Intelligence Scale, WMS- - Wechsler Memory Scale, WCST – Wisconsin Card Sorting Test.

| **Author** | **Study characteristics (Retrospective/Prospective)** | **Nº of patients included (mean age ± standard deviation)** | **Stroke type** | **Stroke location** | **Timing of evaluation after stroke** | **Time evaluation protocol** | **Main results** | **Neurological examination** | **Neuropsychological examination (tests performed)** |
| --- | --- | --- | --- | --- | --- | --- | --- | --- | --- |
| Gooch, Cynthia M. et al. (2009)(16) | Retrospective | 10 patients (59±11)  17 healthy controls (56±11) | 9 ischaemic stroke, 1 haemorrhagic stroke | Cerebellum (6 RH; 4 LH) | 6.3±5.3 years | Prospective timing (estimation, production, reproduction, temporal discrimination) | Lesions in middle, superior cerebellum and dentate nucleus underestimated time intervals and overproduced and overreproduced time intervals over 1 s, no impairment in temporal discrimination.  Left side lesions had more impairment on time tasks. | Normal neurological examination | Normal (MMSE) |
| Harrington, Deborah L., et al. (2004)(18) | Retrospective | 21 patients (58.6±12.2)  30 healthy controls (61.3±10.5) | Ischaemic stroke | Cerebellum (10 RH - 4 superior portion, 6 inferior portion; 11 LH - 5 superior portion, 6 inferior portion) | Right cerebellar stroke - mean 4,6 years±8.1  Left cerebellar stroke - mean 2,7 years±3.8 | Prospective timing (interval comparison and interval reproduction) | Superior cerebellar lesions had impaired performance on time interval reproduction tasks | No data | 10% had impaired performance on digit span from WAIS-R; >40% impaired on TMT-A and 20-30% impaired on TMT-B |
| Malapani C., et al. (1998)(17) | Retrospective | 8 patients (51.3) | 7 ischaemic stroke, 1 haemorrhagic stroke (superior cerebellar artery aneurism rupture) | Cerebellum (2 RH, 3 LH, 3 cerebellar vermis) | 3-6 months | Prospective timing (interval reproduction) | Cerebellar hemispheric lesions had impaired interval reproduction in the seconds range | 3 patients had motor cerebellar syndrome and dysarthria | Normal (WAIS IQs, MMSE, Mattis scale of dementia, CVLT, Grober and Buschke test, WCST and Stroop test, Montgomery and Asberg Depression Rating) |
| Mangels, Jennifer et al. (1998)(19) | Retrospective | 14 patients (62.6)  14 healthy controls (66.3) | Ischaemic stroke | Two groups – Prefrontal lobe (cortical and subcortical white matter involving basal ganglia): 5 LH, 2 RH  Cerebellar group: 3 LH, 4 RH | > 6 months | Prospective timing (time interval comparison in sub (400ms) and suprasecond interval (4000ms) ); | Increased time variability on the short duration task and on the long-duration task  Cerebellar lesions exhibited larger difference thresholds than control subjects on both the short- and long-duration tasks  Frontal lobe lesions demonstrated significantly larger difference thresholds on the long-duration  Regardless of stimulus duration, frontal patients were more likely to classify the comparison interval as ‘short’ compared to both the control subjects and cerebellar patients | 4 of the 5 left hemisphere patients had mild dysphasia, the other patient had severe dysphasia  All cerebellar patients but one had mild to moderate motor cerebellar syndrome | Frontal patients were significantly impaired in WAIS-R Information, Vocabulary and Digit-Span subtests;  Cerebellar patients were significantly impaired in WAIS-R Information and Digit-Symbol subsets  Both produced significantly fewer words than controls on the FAS letter fluency task (WAIS-R, WCST) |
| Casini, Laurence et al. (1999)(20) | Retrospective | 13 patients (64.3±11.2)  10 healthy controls (65.2±5.2) | Ischaemic stroke | Two groups - Dorsolateral prefrontal region (cortical and subcortical white matter): 4 LH, 1 RH  Cerebellar: 3 LH, 4 RH, 1 bilateral | No data | Prospective timing (time interval comparison with auditory stimuli in subsecond interval); second paradigm involved dual tasking with time interval and sound frequency comparison | Both frontal and cerebellar patients were more variable in the duration discrimination task when compared to controls;  Performance was worse for patients when they had a dual task paradigm with concurrent temporal and non-temporal discrimination task | Dorsolateral prefrontal region group - 2 patients with mild aphasia and 1 with severe aphasia  All but one cerebellar patient had motor cerebellar syndrome | Patients performed worse on Digit Symbol subtest of the Wechsler Adult Intelligence Scale—Revised and Verbal Fluency Test; cerebellar patients performed worse than frontal patients in Digit Symbol test (WAIS-R; WCST) |
| Basso, Gianpaolo et al. (1996)(24) | Retrospective | One 59-year-old patient  7 healthy controls | Ischaemic stroke | Right temporal-occipital region, thalamus and posterior arm of internal capsule | 2 months | Prospective timing (time interval comparison and time interval production) | Over-estimation of time duration of stimuli on left hemifield | Patient had left hemianopia, left hemiparesis, left visuospatial neglect | No data |
| Bonato, Mario et al. (2016)(25) | Retrospective | 8 neglect patients (61.1±7.8)  6 non-neglect controls (57.4±14.8) | Ischaemic stroke | Diffuse right hemispheric stroke including right cerebellum | 81.7±53 days | Timed responses concerning the ordinal disposition of 6 key moments in a history in relation to a referencial event (3 before and 3 after the referencial event) | Neglect patients had slower timing responses to events that happened before the referential event | 8 patients with left visuospatial neglect (cancellation test, copy drawing and drawing from memory, line bisection tests) | Normal (MSSE, MoCA) |
| Morin, Catherine et al. (2005)(26) | Prospective | 48 patients  [27 LH (47±3)  21 RH (46±3)]  20 healthy controls (47±3) | Ischaemic stroke | Cortical and subcortical regions (lesions  involved more than one cerebral lobe in 39% of RH and  59% of LH lesions) | 3+-2 days | Clock-time estimation and interview duration estimation | Advance errors were significantly greater in RH patients than in either LH patients or control patients  Delay errors tended to be greater in RH patients than in both LH patients and control patients but the difference was not statistically significant  RH and LH patients with only subcortical lesions did not make pathological advance errors.  There were no differences between patients and controls in interview duration estimation. | Mean value of National Institute of Health Stroke Scale (NIHSS) was 4.9+-0.5 in the LH group vs. 7.4+-1.1 in the RH group | Normal (no information concerning neuropsychological protocol) |
| Kumral, Emre et al. (2007)(33) | Prospective | 5 patients (63±10)  30 healthy controls | Ischaemic stroke | Two left thalamus (mediodorsal nucleus) and 3 bilateral thalami (2 with bilateral mediodorsal nucleus and 1 with bilateral mediodorsal nuclus and right anteromedial nucleus) | 1 day | Daily evaluation for time sense and orientation to time, place and person | All patients with disorientation to actual date, inability to estimate the exact time of the day and under or overestimation of the time passed during the examination.  3 patients were disoriented to actual season of the year. | Patients displayed apathy, emotional blunting, somnolence, reduced verbal fluency, vertical gaze palsy, ataxia, inappropriate behaviours, aggressive outbursts | 3 patients with delayed recall verbal and non-verbal memory tests (MMSE, word list learning and delayed recall, Rey– Osterrieth Complex Figure Test copy and delayed recall, and digit spans backwards and forwards) |
| Coslett, H. Bran et al. (2010)(37) | Retrospective | One 54-year-old patient  13 healthy controls (52.6±8) | Ischaemic stroke | Bilateral caudate, putamen and globus pallidus ischaemic lesions | 7 years | Prospective timing (temporal auditory and visual estimation, temporal auditory and visual production, temporal visual and auditory reproduction, temporal interval comparison) at sub-second and supra-second range | Significant over-estimation of time interval comparison in 300ms range, increasead variability in timed tapping of 50 ms. | Normal | Normal (no information concerning neuropsychological protocol) |
| Coslett, H. Bran et al. (2009)(27) | Retrospective | 29 patients (59.6±11.5)  16 healthy controls (56±11) | Ischaemic stroke | Unilateral lesions involving temporal, parietal and frontal lobes, basal ganglia and thalamus (15 LH patients; 14 RH patients) | 6 months | Prospective timing (temporal auditory and visual estimation, temporal auditory and visual production, temporal visual reproduction at supra-second range) | Patients with brain lesions overestimated, underproduced, but accurately reproduced intervals. | 5 patients had aphasia  (aphasia score based on a picture naming task, a verbal repetition task and a lexical comprehension task) | No subject exhibited neglect on line bisection or line cancellation tasks |
| Cappelletti, M et al. (2011)(31) | Retrospective | One 44 year-old patient  12 healthy controls (43.4; range 40-49) | Ischaemic stroke | Cortical and subcortical left medial, lateral and superior parietal lobe, intraparietal sulcus with extension to temporal region | No data | Prospective timing (temporal visual estimation with non-numerical and numerical trials; time interval comparison of visual stimuli) | Significantly underestimated temporal durations when task-irrelevant numerical stimuli were displayed; near chance performance of interval comparison of visual stimuli with numeral stimuli; accurate performance on tasks with non-numerical stimuli | No data | Verbal and non-verbal intellectual functions showed a moderate decline relative to pre-morbid estimates;  Frontal executive functions were impaired;  Calculus and numerical processing impairment. (NART I.Q, WAIS-R verbal I.Q., WAIS-R performance I.Q) |
| Cappelletti, M et al. (2011)(31) | Retrospective | One 62 year-old patient  12 healthy controls (62.8; range 60-68) | Ischaemic stroke | Cortical and subcortical right inferior parietal lobe, right superior temporal lobe, right inferior frontal and lateral prefrontal areas around the Silvian fissure, right insula and the right basal ganglia | No data | Prospective timing (temporal visual estimation with non numerical and numerical trials; time interval comparison of visual stimuli) | Underestimated the duration of temporal intervals in non-numerical and numerical stimuli;  impaired time duration comparison of two stimuli with both numerical and non-numerical stimuli. | No data | WAIS-R suggested a mild degree of intellectual under-functioning only on tests with a non-verbal component. (NART I.Q, WAIS-R verbal I.Q., WAIS-R performance I.Q) |
| Kaski, Diego et al. (2016)(39) | Retrospective | 18 patients (63.7±10.7)  14 healthy controls (62.6±4.7) | 17 ischaemic stroke and 1 haemorrhagic stroke patient | Cortical and subcortical regions of frontal, parietal, insular and occipital lobes | 5.5±2.4 days | Prospective timing (time interval comparison) - self motion duration comparison based on vestibular-dependent estimation  Patients were further tested for spatial orientation performance | Impaired spatial orientation performance patients (n=4) under-estimated leftward motion duration in comparison with rightward motion duration; these patients had right temporoparietal junction damage. | 6 with left hemianopia  4 with extinction, 4 with neglect | 4 patients with a spatial deficit (position bias) |
| Harrington et al. (1998)(23) | Retrospective | 37 patients [18 RH (64.6±12.2)  19 LH (64.3±9.9)]  48 healthy controls  [24 R controls (66.7±8.3)  24 L controls (63.5±9.1)] | Ischaemic stroke | 9 right brain damage and 6 left brain damage patients with lesions anterior to central sulcus; 10 right brain damage and 12 left brain damage patients with lesions posterior to central sulcus | 3.8 years±4.3 years - left brain damage; 4.1years±3.9years - right brain damage | Prospective timing (time interval comparison) | Impaired time interval comparison in 10 right hemisphere lesion patients. Most lesions were posterior (n=7): supramarginal gyrus and rostral portion of angular gyrus. The anterior lesion group with impaired discrimination (n=3) involved premotor area, superior and medium dorsolateral prefrontal gyrus. | Five RH patients with hemiplegia, 4 LH patients with hemiparesis, both groups had somatosensory deficits.  LH patients had  significant impairment on all measures of aphasia evaluation.  Two RH lesion with contralateral hemianopia  Two RH lesion with neglect | Visuospatial impairment in both groups; LH group was  significantly impaired on all measures of language function, although severity of aphasia was not related to performance on the experimental tasks.  [Aphasia - Western Aphasia Battery, Visuospatial - Block Design subtest from WAIS-R] |
| Mella, Nathalie et al. (2019)(50) | Retrospective | 21 patients  [(57.36±16.27) - right insular lesions; (62.70±12.79) - left insular lesions]  24 healthy controls (64.76±13.78) | Ischaemic stroke | 11 right insula stroke patients and 10 left insula stroke patients | 11 days | Prospective timing (verbal time estimation task and time interval comparison) | Right insular patients presented with under-estimation of short time intervals (300-500 ms) with left ear presented stimulus, less temporal sensitivity in right insula lesion patients | Right insular lesion patients had a mean initial NIHSS score of 4.80±2.76.  Those with a left lesion had a score of 5.10±5.32. | No data |
| Rubia K et al. (1997)(28) | Retrospective | 27 patients (age ranging between 35-65 year-old)  No information about control groupo | Ischaemic stroke | Infarction of the  middle cerebral artery of either the left (n = 11) or  the right (n = 16) hemisphere, including one patient with a right-sided thalamic infarction | 2-24 months | Prospective timing (verbal time estimation task, time production task) | 9 right hemispheric lesion patients with significantly accelerated in subjective time compared with controls (over-estimation in verbal time estimation task, under-reproduction in reproduction time task)  10 left hemisphere lesions patients equally split into those showing acceleration and those showing deceleration in apparent time in both time estimation methods  Posterior supralenticular white matter lesions were associated with significant impairment in time estimation | No data | Sustained attention deficit in 12 patients |
| Trojano, Luigi et al. (2017)(41) | Retrospective | 33 patients (67.7±12.1)  28 healthy controls (73.2±5.1) | Ischaemic and haemorrhagic stroke | 19 right hemispheric lesions and 14 left hemispheric lesions  Patients with impaired performance on prospective items (right inferior parietal cortex, rolandic operculum and posterior middle  temporal gyrus)  Patients with impaired performance on retrospective items (right superior middle temporal gyrus, white matter  posterior to the insula) | 3.4 months | Retrospective and prospective timing (QUEST-R protocol) | Brain lesion damage patients have impaired subjective time performance when compared to controls.  5 right lesion patients with impaired prospective timing (3 with exclusive prospective timing impairment).  7 right lesion patients with impaired retrospective timing (5 with exclusive retrospective timing impairment). | Neglect in 7 right hemisphere lesion patients  (Albert’s cancellation test,  star cancellation test, sentence reading test) | Normal  (MMSE, Frontal Assessment Battery and Clock Drawing) |
| Gooch, Cynthia M et al. (2011)(42) | Retrospective | 47 patients (59±11)  16 healthy controls (57±9) | Ischaemic stroke | 22 left lesion patients involving frontal, temporal, parietal and occipital lobes and striatum  25 right lesion patients involving frontal, temporal, parietal and occipital lobes and thalamus | 6.3±5.3 years | Prospective timing (visual interval comparison in 600 and 2000ms) | Patients with significant frontal damage were less accurate and more variable than controls.  Patients with damage to posterior parietal regions were less accurate than controls.  The right precentral gyrus (lateral BA6), the right middle frontal gyrus (BA9), and the right inferior frontal gyrus (BA44) were important for both 600ms and 2000ms.  Left basal ganglia, superior and middle temporal lobe and hippocampus were important for 600ms perceptual timing. | No data | No data |
| Merrifield, Colleen et al. (2010)(40) | Retrospective | One 64 year-old patient  10 controls [9 healthy controls (mean 67.3±5.7) and left brain lesion control (74-year-old)] | Ischaemic stroke | Right parietal cortex and fronto-parietal subcortical deep white matter, right basal ganglia | 4 months | Retrospective timing (nonverbal time estimation task, verbal time estimation task and visual time estimation task) | Under-estimation of time duration on different protocols in 15-60s interval | Severe left hemiparesis and moderate neglect  (star cancellation, figure copying from Behavioral  Inattention Test, line bisection test) | No data |
| Low, Essie et al. (2016)(32) | Prospective | 40 patients (58.4±9.35)  31 healthy control patients (55.07±8.69) | 30 ischaemic stroke patients and 10 TIA patients | 12 left hemisphere stroke, 18 right hemisphere stroke | 1 to 16 months | Retrospective timing (verbal time estimation task) | Under-estimation of time duration in stroke and TIA groups | Neglect present in 3 RH patients | Impairment in digit span forward and backward in LH stroke patients and RH neglect patients  Normal performance in Raven’s Coloured Progressive Matrices |
| Mole, Joe et al. (2017)(36) | Retrospective | One 50-year-old patient  8 controls (4 healthy controls and 4 brain lesion controls with other aetiology) | Ischaemic stroke | Right anteromedial thalamus | 2 years | Retrospective timing (time duration estimation) and prospective timing (reproduction and production of time intervals) | Impairment in time duration estimation and reproduction; same performance in interval production. | Mild left hemiparesis, left upper quadrantanopia and left hemi-hypoesthesia. | Impaired attention, verbal and non-verbal memory, prospective memory, processing speed, dysexecutive functions (RBMT-III, RMT, WMS-III, D–KEFS, TEA, WAIS-IV, BADS) |
| Danckert, James et al. (2007)(21) | Retrospective | 8 patients (68.6±12.7)  14 controls  [8 healthy controls (mean age 73-year-old, range 60-74 years)  6 brain damage controls (66±14.4) | Ischaemic stroke | Right insula, supramarginal gyrus, caudate, putamen and superior temporal gyrus (intersection lesion areas between patients) | 1 - 24 months | Retrospective timing (verbal time estimation task) | Under-estimation of time duration for 15-60s in neglect patients when compared against healthy controls;  under-estimation of time duration for 30-60s in neglect patients when compared against right hemisphere lesions;  under-estimation of time duration for 60s in right hemisphere lesion patients when compared against healthy controls | Neglect was present on all 8 RH lesion patients  Line bisection, cancellation, and figure copying performance) | No data |
| Koch, Giacomo et al. (2002)(22) | Retrospective | One 49-year-old patient  8 healthy controls (45±5) | Ischaemic stroke | Right dorsolateral prefrontal cortex (Broadmann area 46/49) | No data | Retrospective timing (verbal time estimation task) | Under-estimation of time duration for long time durations (>90 s) | Very mild hemiparesis | Normal (short and long-term memory (RWL; Figure of Rey-b, Immediate and Delayed Recall; Digit Span; Corsi Span, Forward and Backward; Verbal Supraspan; Immediate Visual Memory), visuospatial abilities (RPM), attention (TMT), language (Verbal Fluency, Phrase Construction), executive functions (ToL; WCST), praxia (copy of drawings). |
| Hayashi, Ryuichiro et al. (2018)(34) | Retrospective | One 67-year-old patient | Ischaemic stroke | Right dorsomedial thalamus | 1 month | Prospective timing (time interval reproduction and verbal time duration estimation) | Under-reproduction of time; under and over-estimation of time | Mild dysarthria; temporal disorientation | Revised Addenbrooke's Cognitive Examination score 90/100, Frontal Assessment Battery 13/18  (Revised Addenbrooke's Cognitive Examination, FAB, WMS-R, WCST, forward and backwards digit span) |
| Lee, Soon-Tae et al. (2010)(35) | Retrospective | One 33-year-old patient | Ischaemic stroke | Bilateral dorsomedial thalami | 13 years | Retrospective timing (time duration estimation and inquiry of temporal references of episodic and semantic memory) | Impaired time duration and impaired temporal references in episodic and semantic memories | No data | No data |
| Calabria, Marco et al (2011)(29) | Retrospective | 14 patients (70±10.9 in patients without neglect; 60.3±11.1 in neglect patients)  8 healthy controls (67.2±4.5) | 12 ischaemic stroke and 2 haemorrhagic stroke | Right hemisphere patients (involving frontal, temporal, parietal, occipital, basal ganglia and internal capsule) | 151±141 days | Prospective timing (verbal time estimation task) | Neglect patients overestimated time interval estimation; neglect patients were more severely impaired in terms of time discrimination compared to both HCs and right-brain damaged patients without neglect | Neglect present in 6 patients  (line bisection, cancellation, drawing and reading task) | Normal (MMSE) |
| Montalembert, M et al (2012)(30) | Retrospective | 8 patients (53.6±11.2)  20 healthy controls (46±16.4) | 3 ischaemic stroke and 5 haemorrhagic stroke | Right temporal, occipital, parietal; one patient with internal capsule and thalamic lesion | 80.4 ± 91 days | Prospective timing (verbal time estimation task) | Overestimation of time intervals  Less accurate time interval comparison than healthy participants | Neglect and extinction in 7 patients; 1 patient only had neglect  (line bisection and letter cancellation, a reading task, and a drawing copy task) | Normal (Language, apraxia, agnosia) |
