# Supplementary material for: Time perception changes in stroke patients: A systematic literature review
Source: Front Neurol. 2022 Jul 19;13:938367. doi: 10.3389/fneur.2022.938367 (PMC9343772; doi:10.3389/fneur.2022.938367)
Supplement: Supplementary file 3 [file Table_2.docx]

**Supplemental table 2** - Different time perception deficits and respective studies associated.

| **Time perception deficit** | **Studies identified** |
| --- | --- |
| Underestimation of time intervals | Gooch et al. (10 patients) (16), Cappelletti et al. (2 patients) (31), Kumral et al. (5 patients) (33), Morin et al. (48 patients) (26), Kaski et al. (18 patients) (39), Mella et al. (21 patients) (38), Rubia et al. (10 patients) (28), Merrifield et al. (1 patient) (40), Low et al. (40 patients) (32), Danckert et al. (8 patients) (21), Koch et al. (1 patient) (22), Hayashi et al. (1 patient) (34)  Total: 12 studies involving 165 patients (41,7%) |
| Overestimation of time intervals | Basso et al. (1 patient) (24), Morin et al. (48 patients) (26), Kumral et al. (5 patients) (33), Coslett et al. (1 patient) (37), Coslett et al. (29 patients) (27), Rubia et al. (9 patients) (28), Hayashi et al. (1 patient) (34), Calabria et al (14 patients) (29), Montalembert et al. (8 patients) (30)  Total: 9 studies involving 116 patients (27,8%) |
| Impaired performance on time interval reproduction | Harrington et al. (21 patients) (18), Malapani et al. (8 patients) (17), Mole et al. (1 patient) (36)  Total: 3 studies involving 30 patients (7,6%) |
| Impaired time interval duration comparison | Mangels et al. (14 patients) (19), Casini et al. (13 patients) (20), Cappelletti et al. (2 patients) (31), Harrington et al. (10 patients) (23), Mole et al. (1 patient) (36), Lee et al. (1 patient) (35), Gooch et al (47 patients) (42), Calabria et al (14 patients) (29), Montalembert et al. (8 patients) (30)  Total: 9 studies involving 110 patients (26,3%) |
| Impaired subjective time performance | Trojano et al. (12 patients) (41) (3%) |
| Temporal disorientation | Lee et al. (1 patient)(35), Kumral et al. (5 patients) (33)  Total: 2 studies involving 6 patients (2%) |
| Slower timing responses | Bonato et al. (1patient) (57) (0,3%) |
| Under-reproduction of time interval | Hayashi et al. (1 patient) (34) (0,3%) |
| Over-reproduction of time interval | Gooch et al. (10 patients) (16) (2,5%) |
| Over-production of time interval | Gooch et al. (10 patients) (16) (2,5%) |
